# Gene Expression and Functional Annotation of the Human and Mouse Choroid Plexus Epithelium

**DOI:** 10.1371/journal.pone.0083345

**Published:** 2013-12-31

**Authors:** Sarah F. Janssen, Sophie J. F. van der Spek, Jacoline B. ten Brink, Anke H. W. Essing, Theo G. M. F. Gorgels, Peter J. van der Spek, Nomdo M. Jansonius, Arthur A. B. Bergen

**Affiliations:** 1 Department of Clinical and Molecular Ophthalmogenetics, the Netherlands Institute for Neuroscience (NIN), Royal Netherlands Academy of Arts and Sciences (KNAW), Amsterdam, The Netherlands; 2 Department of Bioinformatics, Erasmus University Medical Center, Rotterdam, The Netherlands; 3 Department of Ophthalmology, University of Groningen, University Medical Center Groningen, Groningen, The Netherlands; 4 Department of Ophthalmology, Academic Medical Centre (AMC), Amsterdam, The Netherlands; 5 Department of Clinical Genetics, Academic Medical Centre (AMC), Amsterdam, The Netherlands; Nathan Kline Institute and New York University School of Medicine, United States of America

## Abstract

**Background:**

The choroid plexus epithelium (CPE) is a lobed neuro-epithelial structure that forms the outer blood-brain barrier. The CPE protrudes into the brain ventricles and produces the cerebrospinal fluid (CSF), which is crucial for brain homeostasis. Malfunction of the CPE is possibly implicated in disorders like Alzheimer disease, hydrocephalus or glaucoma. To study human genetic diseases and potential new therapies, mouse models are widely used. This requires a detailed knowledge of similarities and differences in gene expression and functional annotation between the species. The aim of this study is to analyze and compare gene expression and functional annotation of healthy human and mouse CPE.

**Methods:**

We performed 44k Agilent microarray hybridizations with RNA derived from laser dissected healthy human and mouse CPE cells. We functionally annotated and compared the gene expression data of human and mouse CPE using the knowledge database Ingenuity. We searched for common and species specific gene expression patterns and function between human and mouse CPE. We also made a comparison with previously published CPE human and mouse gene expression data.

**Results:**

Overall, the human and mouse CPE transcriptomes are very similar. Their major functionalities included epithelial junctions, transport, energy production, neuro-endocrine signaling, as well as immunological, neurological and hematological functions and disorders. The mouse CPE presented two additional functions not found in the human CPE: carbohydrate metabolism and a more extensive list of (neural) developmental functions. We found three genes specifically expressed in the mouse CPE compared to human CPE, being *ACE*, *PON1* and *TRIM3* and no human specifically expressed CPE genes compared to mouse CPE.

**Conclusion:**

Human and mouse CPE transcriptomes are very similar, and display many common functionalities. Nonetheless, we also identified a few genes and pathways which suggest that the CPE between mouse and man differ with respect to transport and metabolic functions.

## Introduction

The choroid plexus epithelium (CPE) is a single neural cell layer, which folds itself into a cauliflower-like structure, protruding in the lateral, third, and fourth brain ventricles. On the basolateral side, the CPE lines vascular stroma, while the apical side faces the brain ventricles (reviewed in [1]). The CPE contains tight junctions and forms the blood-CSF barrier (outer blood brain barrier) that prevents passive diffusion of (large) molecules between the CSF and the blood [2], [3]. The CPE produces the cerebrospinal fluid (CSF) and actively transports various molecules from the plasma into the CSF and removes other molecules from the CSF [4]–[8]. The CPE also synthesizes and secretes proteins [9], [10]. Hence, the CPE plays a crucial role in the fluid pressure and balance in the brain ventricles, metabolism of the brain, cellular functions of neurons, immunological and inflammatory process, neurosignaling, neuroprotection after ischemia, and neurodegeneration. The CSF also forms a hydraulic cushion for the brain. Since the CPE is the gate-keeper between the blood and CSF, the tissue is also an interesting target for drug delivery into the CNS [11]. The balance between CSF production and drainage determines the intracranial pressure (ICP). In human, the CSF is primarily reabsorbed into the venous blood through the lymphatic system along the olfactory and optic nerves and through the arachnoid villi along the superior sagittal sinus (reviewed in [12]).

The CPE has been implicated in several neurological disorders, including inflammatory and infectious diseases [13]–[15], trauma [16], ischemic events [17], malignancies [18], Alzheimer's disease (AD) [19], [20] and multiple sclerosis (MS) [21]. In AD, there is stacking of β-amyloid in the CPE cells, the CPE cells have impaired mitochondrial function and increased oxidative stress [19], [20]. Both the production and outflow of CSF decline in AD. This results in a decreased CSF turnover and a changed CSF composition [22]–[24]. Harmful molecules may than accumulate in the CSF and disrupt brain homeostasis. In a subset of AD patients, an impaired blood-CSF barrier was found, which could increase the amount of harmful molecules in the CSF [25]. In MS, CPE cells showed persisting immune activation with immunostaining for T lymphocytes, HLA-DR, and VCAM-1 [21]. Malignancies can originate from the CPE and are classified as follows: CPE papilloma (grade I), atypical CPE papilloma (grade II), CPE carcinoma (grade III), and CPE adenoma [18]. Interestingly, there are indications that the CPE may also play a role in diseases at the periphery of the brain, such as the ocular disorder primary open angle glaucoma (POAG), via an altered CSF-pressure dynamics [26]–[30]. POAG is a neurodegenerative disease of the optic nerve. A major risk factor for POAG is an increased intraocular pressure (IOP), but recent evidence suggests that a decreased ICP may be a risk factor as well [26]–[30]. Consequently, damage to the optic nerve in POAG may be caused by the net pressure force on the optic disk, which is determined by the difference between the IOP and ICP (reviewed in [31]).

Human diseases, like AD, MS and POAG, are frequently studied in transgenic mouse models to understand the *in vivo* biological effect of genetic variation and to develop experimental therapies. As a first step towards constructing transgenic mice that can serve as a model for disturbances in the CPE and CSF formation, it is important to know whether a gene or function of interest is similarly expressed between species. In order to answer the question whether the mouse forms a good animal model to study human CPE related disorders, we need to know the similarities and differences in human and mouse CPE transcriptomes. Consequently, we determined and compared the gene expression profiles and functional annotation of the human and mouse CPE using microarray hybridizations and the knowledge database Ingenuity. We also made a comparison with previously published human and mouse CPE gene expression datasets. In this study, we aimed to answer the following three questions: (1) What is the functional annotation of the human and mouse CPE? (2) Are orthologous genes similarly expressed in human and mouse CPE? (3) Are there human or mouse specifically expressed CPE genes?

## Methods

### 1. Ethics statement

The human CPE material was obtained from the Netherlands Brain Bank (NBB) (Amsterdam, the Netherlands). In accordance with the international declaration of Helsinki, the NBB obtained permission from the donors for brain autopsy and the use of clinical information for research purposes. All procedures of the NBB have been approved by the ethics Committee of VU University Medical Center (Amsterdam, the Netherlands). The human data were all analyzed anonymously.

The study on mouse brain material was carried out in strict accordance with the recommendation in the Guide for the Care and Use of Laboratory Animals under Dutch law, which is in accordance with the international declaration of Helsinki. The protocol was approved by the Committee on Ethical of Animal Experiments of the Netherlands Institute for Neuroscience (NIN), Royal Dutch Academy for Science (KNAW), the Netherlands.

### 2. Gene expression of the healthy human and mouse CPE

We collected seven human CP samples from the lateral ventricles of the human brain. We processed the tissues between 6–24 hours post-mortem time. All tissues were processed anonymously. All donors were male and donor age varied between 51 and 73 years. The donors had no history of any brain disease and post-mortem analysis of the brain revealed no amyloid plaques or tangles (Braak stage zero).

Mouse CPE was obtained from three mouse brains of healthy 18–21 weeks old C57BL/6 mice. We selected adult human donors and adult mice in order to make the comparison as reliable as possible. The C57BL/6 mice (Harlan, Venray, the Netherlands) were raised in a room with a temperature around 21°C, on a 12∶12-h light-dark cycle, and fed with standard pellet laboratory chow and water ad libitum. By the age of 18 to 21 weeks, they were anesthetized with CO_2_/O_2_ and sacrificed with cervical dislocation. The brains were embedded in Tissue Tec OCT, snap-froze in liquid nitrogen and stored at −80°C until use.

We cut out selectively the CPE cells of the human and mouse brain cryosections with laser dissection microscopy in order to exclude contamination with adjacent tissues, like the vascular stroma (PALM Carl Zeiss, Microlaser Technologies AG, Germany). For the human CPE, we cut out 15 cryosections of 10 µm. For the mouse CPE, we cut the whole brain in 20 µm thick vertical slides and selectively laser dissected the whole CPE from the lateral ventricles. We used Cresyl Violet staining to identify the CPE cells. RNA isolation, amplification, and labeling procedures were the same as previously described [32]. Quality of tRNA was checked with BioAnalyzer assay (Agilent Technologies, Amstelveen, the Netherlands, RNA 6000 Pico Kit). RNA integrity numbers (RIN's) of human CPE tRNA ranged from 5.9 to 7.5 (average 6.4) and of the mouse CPE tRNA from 6.5 to 7.9 (average 7.3). We used a common reference study design with RNA from, respectively, human and wild type mouse retinal pigment epithelium/choroid as the common reference. We hybridized seven human and three (genetically homogeneous) mouse CPE samples against their common reference samples on, respectively, catalogue human 4×44K and mouse 1×44K microarrays (Agilent Technologies, Amstelveen, the Netherlands). See Janssen et al. [32] for a detailed description of the laser dissection procedures, RNA processing and microarray procedures.

We submitted our microarray data to Gene Expression Omnibus (GEO) database and the accession number is GSE49974.

The microarray image files were analyzed and processed by Agilent Feature Extraction Software (Agilent Technologies, version 9.5.3.1) and mean intensities (log2) were given to the spots. In R (version 2.14.0 for Windows, R Development Core Team, 2009), we normalized the mean intensities between arrays (NormalizeBetweenArrays with method ‘aquantile’). We ranked the genes by expression level and assigned percentile ranks (P) [33]. Next, we formed four sub-datasets: high expression (expression >90^th^ P), moderate expression (50–90^th^ P), low expression (10–50^th^ P) and very low expression (<10^th^ P). This means that a gene in the high expression sub-dataset (>90^th^ P) has an expression intensity that falls into the highest 10% intensity values of the microarray, whereas a gene in the very low expression sub-dataset (<10^th^ P) has an expression intensity in the lowest 10% intensity values of the microarray (methodology according to Booij [33]; Janssen [32]).

### 3. Functional annotation of human and mouse CPE

We functionally annotated the “Human and Mouse High CPE expression sub-datasets” with the knowledge database IPA (Ingenuity Systems version 14855783; date of analysis between December 2012–April 2013; www.ingenuity.com). We selected the highly expressed genes for analysis since these genes are most likely of biological importance [33]. IPA, commonly called “Ingenuity” is a knowledge database and bioinformatics program aimed at the interpretation of gene expression data across species. An Ingenuity ‘core analysis’ typically generates biological functions, canonical pathways and functional molecular networks. We set the relevant statistical parameters in Ingenuity to Benjamini-Hochberg multiple testing correction and statistical significance was set from p-values<0.001. For the functional molecular networks, we set Ingenuity software to build networks with maximum 35 molecules and a maximum of 25 networks. For more detailed information on Ingenuity see www.Ingenuity.com. We also compared the human and mouse functional annotation of the different CPE gene expression sub-datasets (high, moderate, low and very low expression sub-datasets). We did this by making a list of all significant functions attributed to, for example, the “Human High CPE expression sub-dataset” and the “Mouse High CPE sub-dataset”. Next, we compared these lists, looking for potential overlap or differences between assigned functions.

### 4. Human or mouse specifically expressed CPE genes

**Figure 1:**
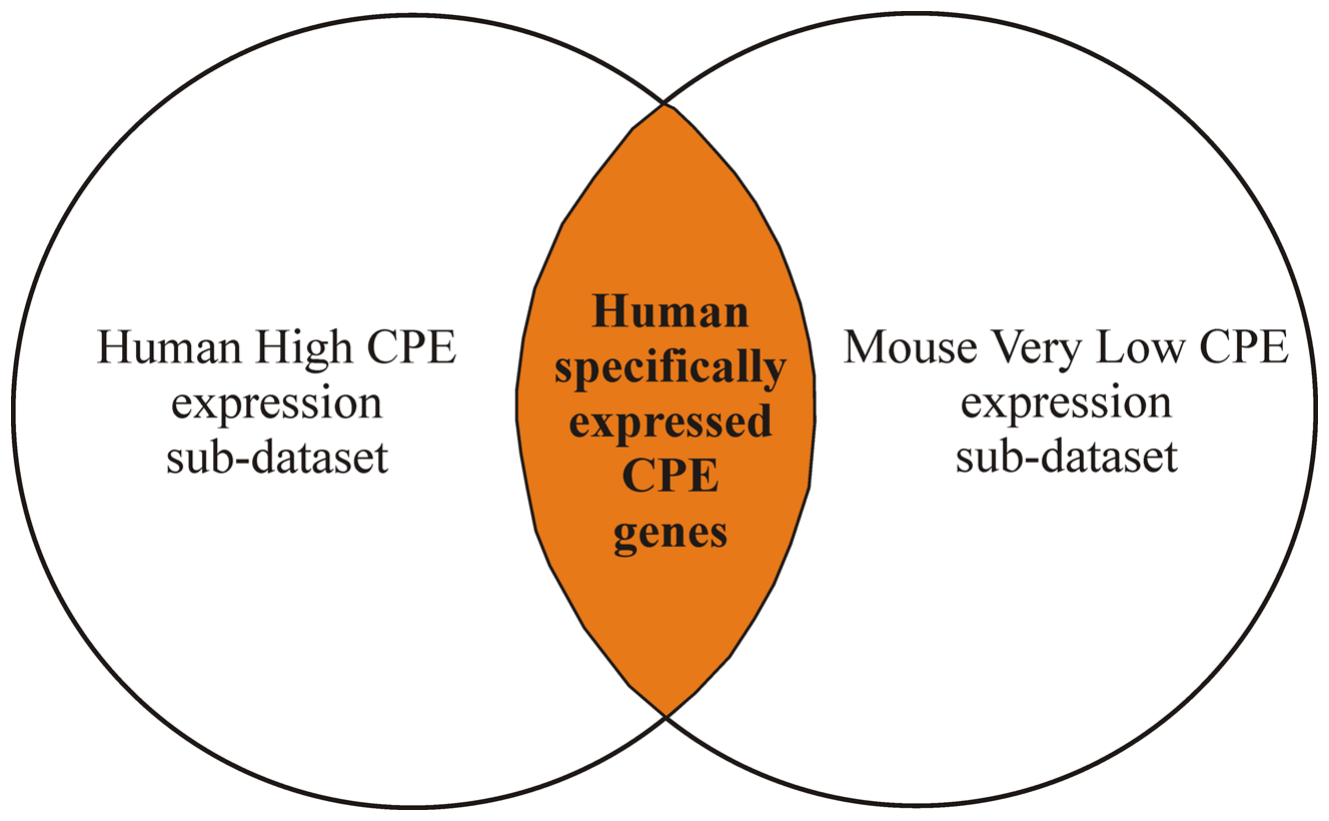
Human specifically expressed CPE genes. Schematic graph to illustrate the determination of the human specifically expressed CPE genes compared to mouse CPE. We compared the “Human High CPE expression sub-dataset (>90^th^ P)” with the “Mouse Very Low CPE expression sub-dataset (<10^th^ P)” and selected the common genes that showed consistent hybridization signals and had a formal GenBank NM number. These genes, indicated in the orange overlapping area, are called the human specifically expressed CPE genes.

To discover human CPE genes that were specifically expressed compared with the mouse CPE, we compared the “Human High CPE expression sub-dataset (>90^th^ P)” with the “Mouse Very Low CPE expression sub-dataset (<10^th^ P)” (Figure 1). We only compared orthologous genes of human and mouse with the *same gene name annotation* in Ingenuity, with the function “compare data”. We corrected for potential gene duplicates and selected only oligos representing genes with a formal GenBank NM Accession number. Array duplicate features that showed inconsistent hybridization signals were also removed from the comparison. We consider these genes that are highly expressed in human CPE, and very lowly expressed in the mouse CPE, to be specifically expressed in the human CPE.

Similarly, a comparison of the “Mouse High CPE expression sub-dataset (>90^th^ P)” with the “Human Very Low CPE expression sub-dataset (<10^th^ P)” yielded the genes that were specifically expressed in the mouse CPE compared to human CPE.

### 5. Confirmation of gene expression data by RT-PCR

We confirmed our microarray data with semi-quantitative RT-PCR (sqRT-PCR). For sqRT-PCR, we selected five genes that were substantially higher expressed in human CPE than in mouse CPE. This meant that the genes were highly (>90th P) expressed in human CPE and lowly (0–50th P) in mouse CPE. These five genes were *ANKK1*, *FBP2*, *ILDR1*, *NELL2*, and *THEM5*. We also selected five genes that were highly expressed in both human and mouse CPE (*ATP1B1*, *IGFBP7*, *NPC2*, *RPL13*, and *SLC13A4*), and five genes that were substantially higher expressed in mouse CPE compared to human CPE (*ACE*, *LBP*, *PON1*, *SLC7A10*, and *TRIM3*). The procedure of the sqRT-PCR was previously described by Booij et al. [34]. In short, sqRT-PCR was carried out using intron spanning primers on cDNA from laser dissection microscopy derived samples of human and mouse CPE. Since we worked with human post-mortem donor material which consists of intrinsically shorter and partly degenerated RNA fragments, we could not use catalogues RT-PCR assays, and we had to generate primers near the 3′ end of the gene. Because of this sub-optimal primer design, PCR was not always optimal and some cDNAs showed no PCR product at all. The primer sequences used are available on request.

### 6. Comparison with other gene expression datasets of the CPE from the literature

We compared our gene expression of the healthy human and mouse CPE with those of previously published CPE gene expression datasets. Obviously, these literature microarray studies had their own strengths and limitations in terms of method of sample isolation, RNA quality control and processing, number features screened, data analysis, and interpretation. We transferred the literature datasets into our own data structure to make all data and studies maximally comparable. We used literature gene expression database of healthy human CPE (GSE14098 [35]) and three of healthy mouse CPE (GSE23714 [36]), GSE37098, GSE33009 [37]). For each of these gene expression datasets, we selected the highly expressed genes (>90th percentile), as described above (Methods paragraph 2). We functionally annotated these highly expressed sub-datasets and compared the analyses with ours.

## Results

### 1. Gene expression of human and mouse CPE

Our gene expression data of the human and mouse CPE can be found in the GEO database (GSE49974). For both the human and mouse CPE, we ranked the genes in four different sub-datasets: high, moderate, low and very low expression (see Methods). We selected the “Human and Mouse High CPE expression sub-datasets” (>90^th^ P) (number of genes, respectively, n = 3361 and n = 3699) for further functional annotation and determined potential human and mouse specifically expressed CPE genes. We also analyzed genes that were highly (>90^th^ P) expressed in one species and simultaneously low in the other species and we studied the genes of the very low CPE expression sub-datasets (<10^th^ P; number of genes human n = 3179 and mouse n = 3583). These two latest analyses did not lead to additional interesting information and are therefore not shown.

### 2. Functional annotation of our “Human High CPE expression sub-dataset”

Functional annotation of gene expression datasets using the Ingenuity knowledge database yields *biological functions, canonical pathways, and functional molecular networks*.

For our “Human High CPE expression sub-dataset” the major seven *biological functions* were: (1) neurological function and disease, including Alzheimer's disease, Parkinson's disease, Huntington's disease, Leigh syndrome, mitochondrial cytopathy, hydrocephalus, neuromuscular disease, tauopathy, gliosis, and nervous system development; (2) immunological and inflammatory function and disease, including viral infection pathways, rheumatoid arthritis, psoriasis, and auto-immune disease; (3) developmental function and disease, including adhesion of embryonic cells, proliferation of fibroblasts and muscle cells, adhesion of kidney cells, and morphology of neuroglia; (4) hematological function and disease, including hematopoiesis, anemia, erythrocytosis, Fanconi anemia, and Diamond-Blackfan anemia; (5) free radical scavenging; (6) energy production; and (7) basic cellular (dys)functions, including gene expression, protein synthesis and degradation, cell cycle, molecular transport, cell-to-cell signaling, cellular movement, and cancer.

Functional annotation of our “Human High CPE expression sub-dataset” also yielded statistically significant *canonical pathways*. We subdivided those in seven groups: (1) actin cytoskeletal function; (2) epithelial junctions; (3) vesicle mediated transport; (4) oxidative stress pathways; (5) immunological function; (6) endocrine signaling and metabolism; and (7) basic cellular (dys)functions (Table S1).

**Figure 2:**
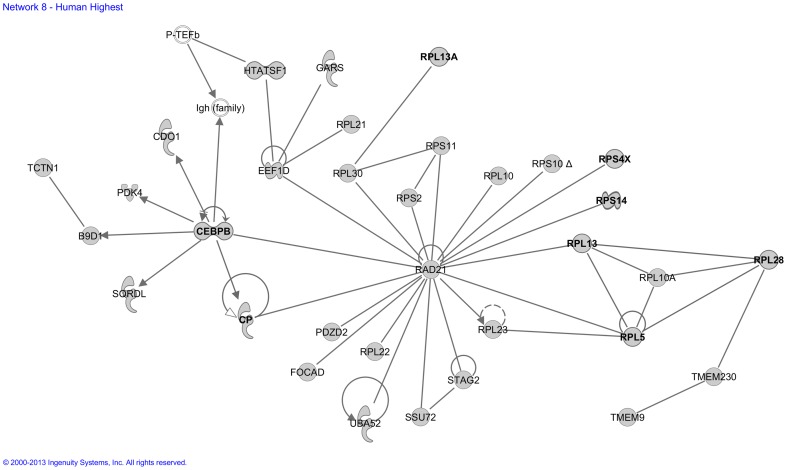
Molecular network generated by the Ingenuity software from the “Human High CPE expression sub-dataset”. Example of a molecular network generated by Ingenuity from the “Human High CPE expression sub-dataset (>90^th^ P)” of the microarray analysis. Grey symbols represent genes that are highly expressed in the human CPE. Transparent entries are molecules inserted by the knowledge database. Abbreviations of gene names are according to standard abbreviations used in GenBank. Solid lines between molecules indicate direct physical or functional relationships between molecules (such as regulating and interacting protein domains); dotted lines between molecules indicate indirect functional relationships (such as co-regulation of expression of both genes in cell lines). The genes *CEBPB*, *CP*, *RPL5*, *RPL13*, *RPL28*, and *RPS14* were previously implicated in microcytic and Diamond-Blackfan anemia and myelodysplastic syndrome and the genes *RPL5*, *RPL13*, *RPL13A*, and *RPS4X* were previously implicated in Parkinson's disease and motor neuropathy (these genes are indicated bold in the Figure). The main functionalities given by Ingenuity for this entire molecular network are ‘Cancer and hematological and metabolic disease’.

**Figure 3:**
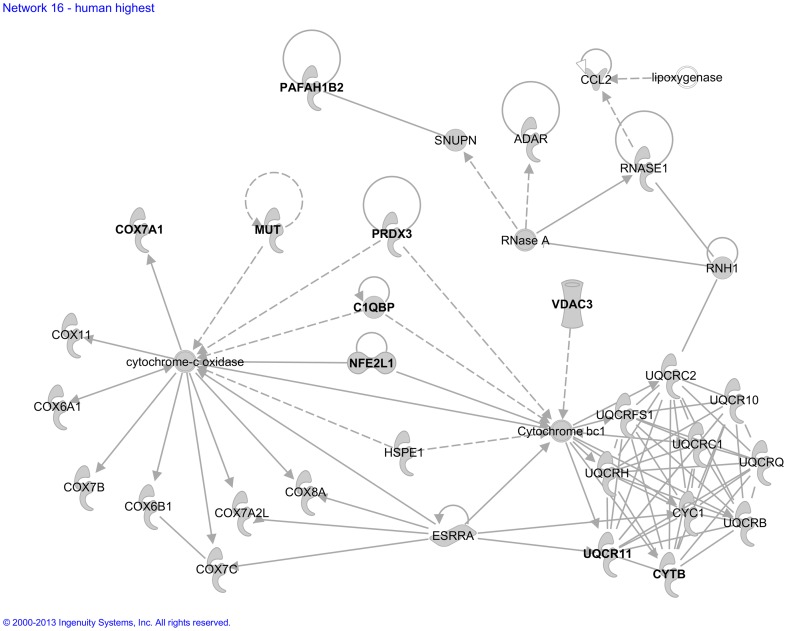
Molecular network generated by the Ingenuity software from the “Human High CPE expression sub-dataset”. Example of a molecular network generated by Ingenuity from the “Human High CPE expression sub-dataset (>90^th^ P)” of the microarray analysis. For explanation of symbols on the diagrams see legend Figure 2. This network contains several genes that code for proteins involved in transport of specific biomolecules, namely *CYTB* and *UQCR11* (transport of H+), *MUT* and *NFE2L1* (transport of glutathione and carnitine), *VDAC3* (transport of adenine) and *C1QBP* (transport of hyaluronic acid and lactic acid). The genes mentioned are indicated bold in the Figure. The main functionalities given by Ingenuity for this entire molecular network are ‘Hereditary disorder, metabolic disease and molecular transport’.

We next analyzed the 25 *functional molecular networks* that Ingenuity assigned to our “Human High CPE expression sub-dataset” (Table S2). Functional annotation of these networks included developmental functions and hereditary disorders, cellular transport functions and diseases in hematological, metabolic, connective tissue, cardiovascular, and endocrine systems. Figure 2 presents, as an example, network 8, which contained several genes involved in hematological and neurological disorders. Another example is network 16 (Figure 3), which contained many entries involved in transport.

### 3. Functional annotation of our “Mouse High CPE expression sub-dataset”

The functional annotation of our “Mouse High CPE expression sub-dataset” yielded similar *biological functions* compared to the human analysis (described above). Furthermore, we found unique biological mouse CPE functions, not present in the “Human High CPE expression sub-dataset”, being (A) metabolic function and disease, including carbohydrate metabolism, lipid metabolism, aciduria, mitochondrial disorder hyperglycemia, hyperinsulinism, hypoglycemia, and amyloidosis; and (B) a more extensive list of developmental functions, including development and morphology of the brain, eye, and cardiovascular system, as well as fusion of fetal membranes and embryo-size.

**Figure 4:**
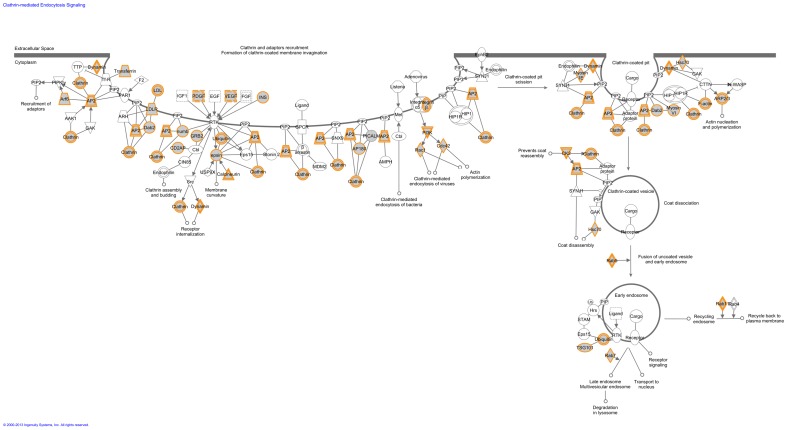
Clathrin-mediated endocytosis signaling pathway identified by the Ingenuity software in the “Mouse High CPE expression sub-dataset”. This is one of the canonical pathways that contained statistically significantly more genes than expected by chance in the group of genes that are highly expressed in the mouse CPE. Gray fields indicate their presence in the “Mouse High CPE expression sub-dataset (>90^th^ P)”; uncolored genes are added by the Ingenuity software to show the entire standard pathway. Solid lines between molecules indicate direct physical relationships between molecules (such as regulating and interacting protein domains). Abbreviations of gene names are according to standard abbreviations used in GenBank. As shown, the majority of genes of this pathway are highly expressed in the mouse CPE.

For our “Mouse High CPE expression sub-dataset”, we identified the same *canonical pathways* as we found in the “Human High CPE expression sub-dataset”. In addition, we made the following observations: First, there were far more statistically significant canonical pathways in our “Mouse High CPE expression sub-dataset”. All these additional pathways were categorized into the seven groups mentioned above (see functional annotation of the “Human High CPE expression sub-dataset”), except for “Mouse embryonic stem cell pluropotency”. Furthermore, the vesicle mediated transport pathway “Clathrin-mediated endocytosis signaling” was only statistically significant in our “Mouse High CPE expression sub-dataset” (Figure 4), whereas “Caveolin-mediated endocytosis signaling” was statistically significant in both our “Human and Mouse High CPE expression sub-datasets”. Finally, our “Mouse High CPE expression sub-dataset” displayed more metabolic canonical pathways, for example “Fatty acid beta-oxidation I” and “TCA cycle II”. Table S1 presents an overview of all statistically significant canonical pathways of the “Human and Mouse High CPE expression sub-datasets”.

**Figure 5:**
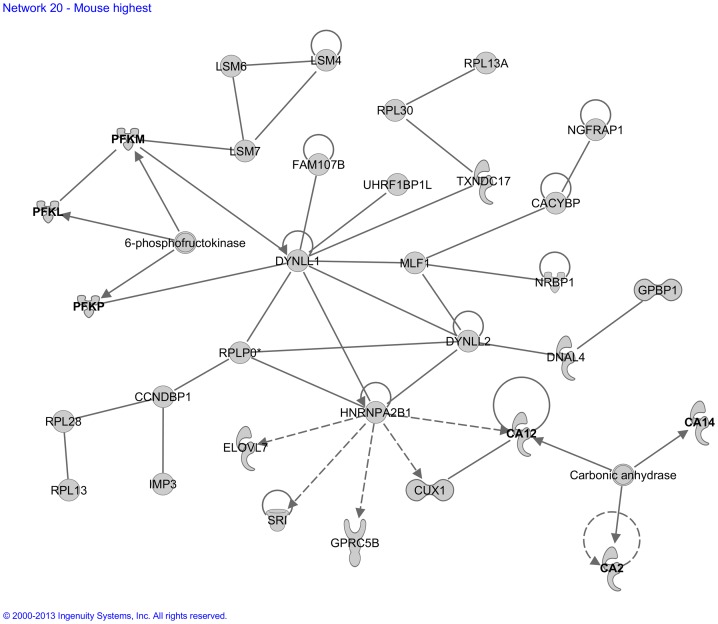
Molecular network generated by the Ingenuity software from the “Mouse High CPE expression sub-dataset”. Example of a molecular network generated by Ingenuity from the “Mouse High CPE expression sub-dataset (>90^th^ P)” of the microarray analysis. For explanation of symbols on the diagrams see legend Figure 2. The genes *PFKL*, *PFKM* and *PFKP* were previously implicated in glucose metabolism and *CA2*, *CA12* and *CA14* were attributed to diseases with disturbed lipid and/or glucose metabolism and are involved in CSF production. These genes are indicated bold in the Figure. The main functionalities given by Ingenuity for this entire molecular network are ‘Carbohydrate metabolism, ophthalmic disease and metabolic disease’.

**Figure 6:**
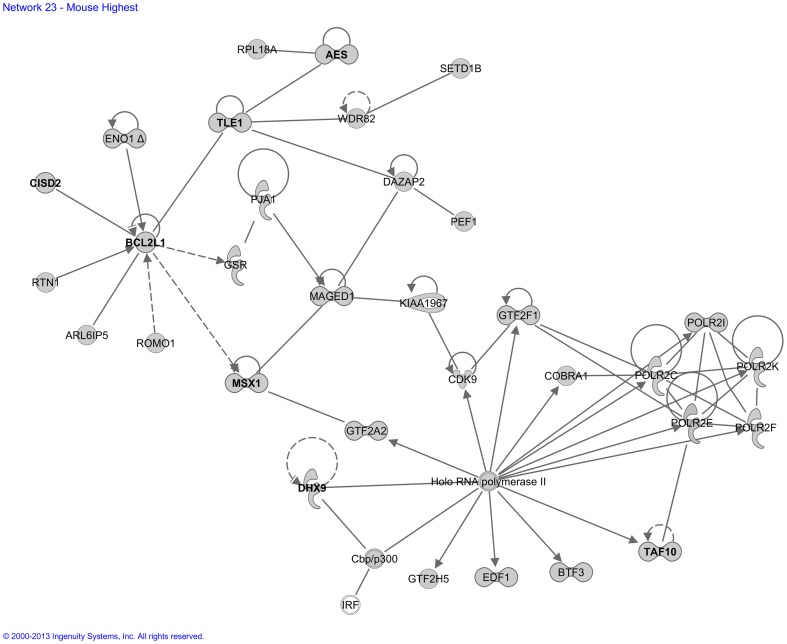
Molecular network generated by the Ingenuity software from the “Mouse High CPE expression sub-dataset”. Example of a molecular network generated by Ingenuity from the “Mouse High CPE expression sub-dataset (>90^th^ P)” of the microarray analysis. For explanation of symbols on the diagrams see legend Figure 2. In this network there are genes involved in maturation of embryonic stem cells, generation of dopaminergic neurons and survival of RPE cells (*BCL2L1*), size of the eye (*AES, TLE1*), development of neural crest (*MSX1*), morphogenesis of cartilage tissue and middle ear (*MSX1*), and apoptosis of the embryoblast (*TAF10*). These genes are indicated bold in the Figure. The main functionalities given by Ingenuity for this entire molecular network are ‘Gene expression, embryonic and organ development’.

For our “Mouse High CPE expression sub-dataset”, Ingenuity built *25 functional molecular networks* with functionalities including development and hereditary disorders, metabolism of glucose and lipids, hematological disease, neurological disease, connective tissue disease, and ophthalmic disease (Table S3). An example is network 20 (Figure 5), which contained several genes involved in glucose metabolism. Another example is network 23 (Figure 6), which contained many genes previously implicated in developmental processes.

### 4. Human and mouse specifically expressed CPE genes

#### 4.1. Human specifically expressed CPE genes

Following our strict selection criteria, we compared our “Human High CPE expression sub-dataset” with our “Mouse Very Low CPE expression sub-dataset”: we did not find any gene(s) specifically expressed in the human CPE compared to the mouse CPE.

#### 4.2. Mouse specifically expressed CPE signature genes

We found three genes that were specifically expressed in the mouse CPE compared to human CPE, namely *ACE*, *PON1* and *TRIM3* (Table 1).

**Table 1:** Mouse specifically expressed CPE genes compared to human CPE.

Gene Name	Systemic name Mouse	Systemic name Human
*ACE*	NM_009598	NM_152831
*PON1*	NM_011134	NM_000446
*TRIM3*	NM_018880	NM_006458

### 5. Confirmation of gene expression data by PCR

**Figure 7:**
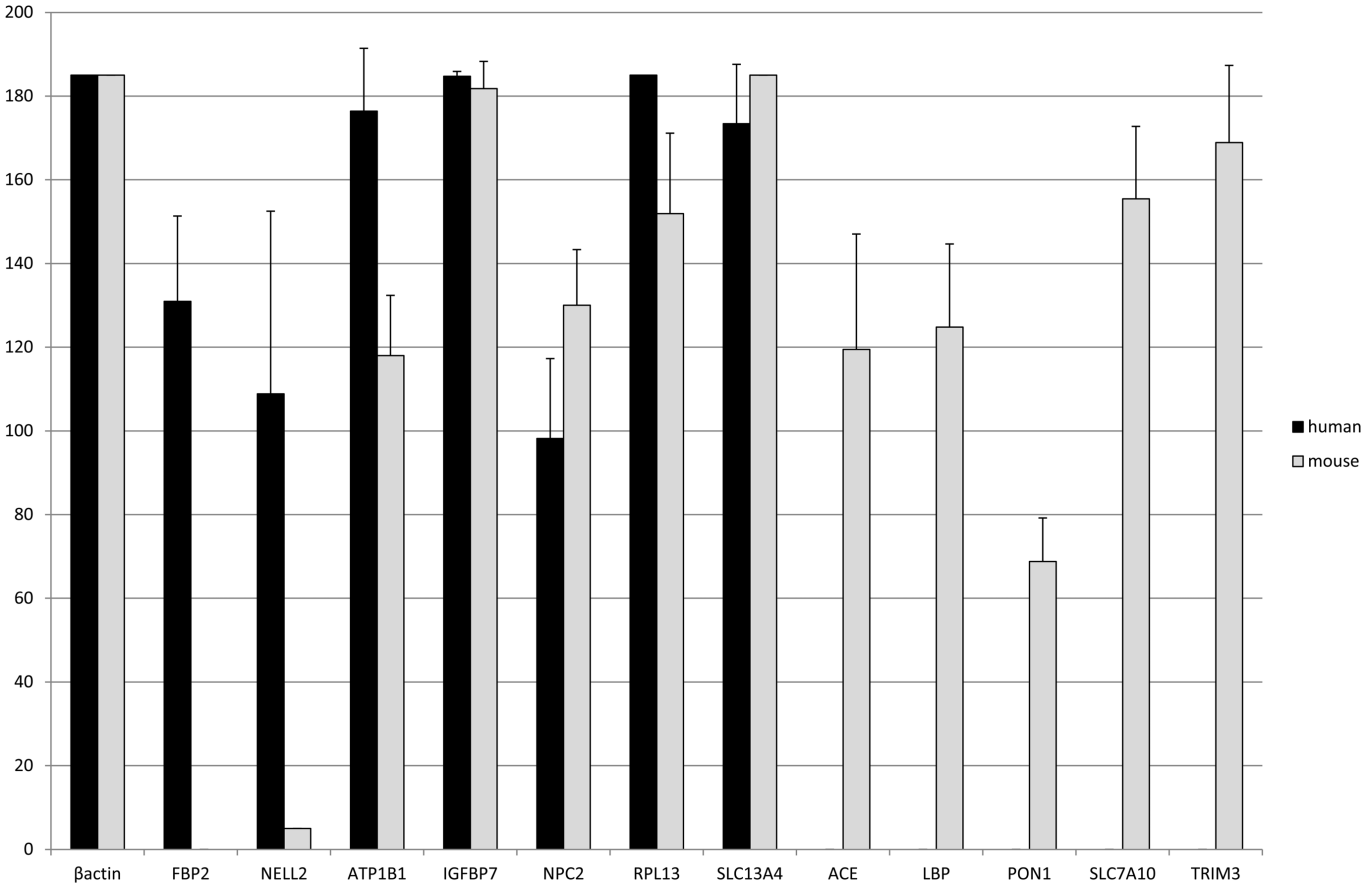
Confirmation of microarray results by sqRT-PCR. Beta actin, a household gene, was used to normalize gene expression in the human and mouse CPE. With semi quantitative RT-PCR (sqRT-PCR), we could confirm our microarray results, namely two genes that were substantially higher expressed in human CPE compared to mouse CPE (*FBP2* and *NELL2*), five genes that were highly expressed in both human and mouse CPE (*ATP1B1*, *IGFBP7*, *NPC2*, *RPL13*, and *SLC13A4*) and five genes that were substantially higher expressed in mouse CPE compared to human CPE (*ACE*, *LBP*, *PON1*, *SLC7A10*, and *TRIM3*). For all genes, positive controls were positive. For example, the primer set for human *ACE* did showed PCR product in the positive control sample of placenta cDNA, and when we performed PCR in human CPE sample, we found no PCR product for *ACE*, indicating that there was hardly any RNA of *ACE* in the human CPE. This was in correspondence of the microarray data. Black bars indicate human CPE expression and grey bars mouse CPE expression. Standard deviations were calculated and indicated in the bars.

For all 14 selected genes (*ANKK1*, *FBP2*, *ILDR1*, *NELL2*, *ATP1B1*, *IGFBP7*, *NPC2*, *RPL13*, *SLC13A4*, *ACE*, *LBP*, *PON1*, *SLC7A10*, and *TRIM3*), the positive controls were indeed positive (not shown). The positive control samples included placenta, cortex, and liver cDNA samples. For 12 genes, we could directly confirm our gene expression data with sqRT-PCR (Figure 7). For *THEM5*, we could not design optimal primers (see Methods section). A few primer sets did not yield PCR product for positive controls of human and mouse CPE. So we excluded this gene from the list. The PCR of the genes *ANKK1* and *ILDR1* were suggestive but not significant confirmative.

### 6. Comparison with other gene expression datasets of the CPE

We compared our gene expression data of the human and mouse CPE with those of previously published CPE gene expression data, namely one healthy human CPE (GSE14098 [35]) and three healthy mice CPE (GSE23714 [36]; GSE37098; GSE33009 [37]). The characteristics of these datasets as well as ours are presented in Table 2. For these additional four gene expression datasets, we also selected the highest expressed genes (>90th P) (see Methods) and we conducted a functional annotation of the sub-datasets in Ingenuity comparable to our own analyses described above.

**Table 2:** Basic characteristics of different human and mouse gene expression datasets.

Species	M/F	Age	n	GEO	Platform	N° genes total	N° genes >90^th^ P
**Human**	**M**	**51–73 y**	**7**	**GSE49974**	**Agilent-014850 Whole Human Genome Microarray 4×44K G4112F (GPL4133)**	**33.469**	**2.722**
Human	?	?	8	GSE14098	Affymetrix Human Genome U133A 2.0 Array (GPL571)	13.384	1.106
**C57BL/6 mice**	**F**	**18–21 wks**	**3**	**GSE49974**	**Agilent-012694 Whole Mouse Genome G4122A (GPL2872)**	**33.542**	**3.467**
C57BL/6 mice	M	8–9 wks	3	GSE23714	Illumina mouseRef-8 v1.0 expression beadchip (GPL6100)	19.077	1.277
C57BL/6J mice	M	3 wks	5	GSE37098	Affymetrix Mouse Gene 1.0 ST Array (GPL6246)	35.558	2.271
Webster mice	F	10 wks	3	GSE33009	Affymetrix Mouse Exon 1.0 ST Array (GPL6096)	23.332	1.741

Basic characteristics of our healthy human and mouse CPE gene expression datasets (bold printed) and those from previously published healthy human and mouse CPE.

Abbreviations: M = male; F = female; y = years; wks = weeks; n = number of sample size; N° = number of; P = percentile.

Within the limitations of each study, there was substantial overlap between our “Human and Mouse High CPE expression sub-datasets” and previously published data. Also their functional annotations per species were highly similar. The three functions that we found in mouse CPE but not in human CPE, were, in part, confirmed (metabolism and more extensive list of developmental functions) and, in part, not (clathrin-mediated endocytosis signaling). For the three specifically expressed mouse CPE genes compared to human CPE, we found high expression of *ACE* and moderate expression of *PON1* and *TRIM3* in all mouse CPE gene expression datasets. We identified low expression of *TRIM3* in the human CPE expression dataset GSE14098; for *ACE* and *PON1*, no additional human CPE data were available in GSE14098. The detailed comparison is summarized in Table 3.

**Table 3:** Comparison of different human and mouse CPE gene expression analysis.

Datasets	Total^1^	Overlap^2^	Funct. Annot.^3^	Metab^4^	Dev^4^	Clath^4^	*ACE* 5	*PON1* 5	*TRIM3* 5
**Human- GSE49974**	**3361**			**−**	**−**	**−**	**VL**	**VL**	**VL**
Human- GSE14098	1106	787 (71%)	h-s	−	−	+	x	x	L
**Mouse-GSE49974**	**3699**			**+**	**+**	**+**	**H**	**H**	**H**
Mouse- GSE23714	1277	1072 (84%)	h-s	+	+	+	H	M	M
Mouse- GSE37098	2271	1450 (64%)	h-s	+	+	+	H	M	M
Mouse- GSE33009	1741	1110 (64%)	h-s	+	+	+	H	M	M

Comparison of our (bold printed) human and mouse CPE gene expressions, functional annotation and specifically expressed genes with those of previously published human and mouse CPE transcriptomes.

^1^ Total numbers of genes in the High Expression (>90th percentile (P)) sub-datasets.

^2^ Comparison of the genes in the >90th P of different datasets from literature with our “Human and Mouse High CPE expression (>90^th^ P) sub-datasets” per species. The number and percentage of overlap is given.

^3^ Comparison of the functional annotation (Funct. Annot); h-s = highly similar.

^4^ Comparison with mouse specific functions. Metab = Metabolism: metabolic function and diseases, including carbohydrate metabolism, lipid metabolism, aciduria, mitochondrial disorder, hyperglycemia, hypoglycemia and amyloidosis. Dev = Development: More extensive list of developmental functions, including development of eye, cornea, cortex, brain and embryo-size. Clath = Clathrin: Clathrin-mediated endocytosis signaling.

^5^ Comparison with specifically expressed genes. We identified three genes that we specifically expressed in mouse CPE compared to the human CPE, namely *ACE*, *PON1* and *TRIM3*. We subdivided the gene expression data of human and mouse CPE from literature also in groups based on percentiles (P): high (H) (>90^th^ P), moderate (M) (50–90^th^ P), low (L) (10–50^th^ P) and very low (VL) (<10^th^ P) and we looked in which of these groups the three genes were expressed in the gene expression databases from literature. Sometimes, no data were available for a gene (indicated with “x”).

## Discussion

In the present study, we investigated and compared the gene expression profiles and functional annotations of healthy human and mouse CPE.

The expression of most of the orthologous CPE genes is highly similar between human and mouse. In contrast with the human CPE, the mouse CPE specifically expressed three genes, namely *ACE*, *PON1* and *TRIM3*.

We found that the functional annotations of human and mouse CPE are highly comparable, with respect to presence of epithelial junctions, transport, energy production, neuro-endocrine signaling, and immunological, neurological and hematological functions and disorders. The mouse CPE also presented two additional functions not found in the human CPE, namely carbohydrate metabolism pathways and a more extensive list of (eye and neural) developmental functions.

### 1. Study design of CPE gene expression

The technical and methodological strengths and limitations of our microarray approach were already extensively discussed elsewhere [33], [38]. In short, the strengths include inclusion of seven different human donor tissues, use of a laser dissection microscope for high cellular specificity and minimal tissue manipulation, large scale analysis using a 44k microarray and a common reference design for normalization between the samples. A limitation is that we used post-mortem human material. To correct for this, we stringently controlled sample and RNA quality (as measured by RNA RIN values) in every step of the protocol and we used standardized catalogue arrays with many internal controls. Furthermore, we focused our analysis of groups of genes with a wide range of expression levels and a single specific function, rather than on individual gene expression levels alone. Another limitation is that our cut of criteria for high and low expression levels are arbitrary.

For the current study, some additional consideration and choices were made. Gene expression profiles of human and mouse CPE were studied on catalogue whole-genome microarrays (Agilent) that were separately designed for human and mouse orthologous genes but do not target exactly the same gene sequence. Therefore, the human and mouse probes have different affinities to their target RNAs and this hampers a *direct* comparison between human and mouse RNA expression data. To solve this problem, we choose to rank the gene expression data and subdivided the total dataset in four different expression groups: high, moderate, low and very low expression, essentially according to Booij et al 2009. For each gene, a rank number according to this subdivision was given, which made comparison of relative gene expression levels between mouse and human possible. By comparing the most extreme sub-datasets, namely the high (>90^th^ P) and very low (<10^th^ P) between species, we could identify physiological relevant differences between human and mouse CPE gene expressions, since these major differences could not be caused by different affinities alone. Furthermore, the three mouse specifically expressed CPE genes compared to human CPE were confirmed with sqRT-PCR. Other strategies with a number of other (dis) advantages exist to compare human and mouse expression data. These were based on the comparison between diseased and healthy human and mouse tissues [39], [40], or the comparison between more than two tissues of human and mouse [41] or on many different tissues together and with the major goal to study overall homology between human and mouse, without tissue-specific questions [42]–[44]. A full methodological description comparing all (dis-)advantages is outside the scope of this paper. However, with our approach healthy human and mouse CPE expression profiles can be compared specifically and in detail.

### 2. Functional annotation of human and mouse CPE

#### 2.1 Common functionalities of human and mouse CPE

The most important common functionalities of the human and mouse CPE, based on our gene expression dataset, were: presence of epithelial junctions, transport properties, energy production and oxidative stress, neuro-endocrine signaling, and immunological, neurological and hematological function and disease.

**Figure 8:**
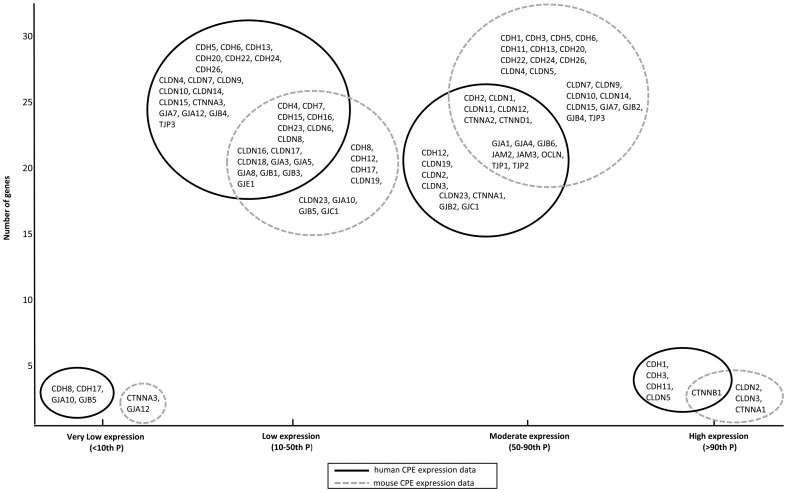
Expression of epithelial junction genes in human and mouse CPE. This diagram illustrates the distribution of expression of epithelial junction genes in human and mouse CPE, subdivided in the four sub-datasets (very low (<10^th^ P); low (10–50^th^ P); moderate (50–90^th^ P); and high (>90^th^ P); see Methods). On the x-axis, the four different expression subgroups are displayed, on the y-axis the number of genes. The black solid lined circles contain epithelial junctions expressed in human CPE expression data and the grey dotted lined circles include those of the mouse CPE. The epithelial junction genes showed overlap in expression category between human and mouse CPE, and none of them showed a difference in expression more than one sub-dataset between human and mouse. For example, the genes CDH1 and CDH3 were highly expressed in human CPE and moderately in mouse CPE, whereas the genes CLDN2 and CLDN3 were highly expressed in mouse CPE and moderately in human CPE and the gene CTNNB1 was highly expressed in both human and mouse CPE.

The expression of genes coding for *epithelial junctions* were highly comparable between human and mouse CPE. The epithelial junction genes showed overlap in expression category between human and mouse CPE, and none showed a difference in expression more than one sub-dataset between human and mouse (Figure 8). Taken together, our data thus suggest that there may be minor expression differences in CPE cell adherence genes between human and mouse. Future, more specific physiological studies should make it clear whether these minor expression differences ultimately translate themselves into high similarities in CPE barrier function between these two species.

We also compared the previously published human and mouse CPE datasets with respect to the expression profiles of the epithelial junctions. These were highly similar with our data. For example, we found high expression of *CTNNA1* and *CTNNB1* in all gene expression datasets, moderate to high expression of *GJA1* and *TJP1* in human CPE and moderate to high expression of *CLDN1* and *CLDN2* in all mouse CPE gene expression datasets.

Obviously, *transporters* play an important role in the CPE. Besides the production of CSF, the CPE also transports a variety of biomolecules and acts as a sink for xenobiotics.

Previously, Brown et al. [45] reviewed the ion-channel proteins in the CPE that are involved in CSF production. We now identified the expression of the genes corresponding to these ion channel proteins in all human and mouse CPE gene expression datasets available to us (Table 4). Overall, the gene expression values were highly comparable, not only between different studies but also between species. Some of these ion-channel genes were highly expressed across all datasets, namely *AQP1, ATP1A1, ATP1A2, ATP1B1*, *ATP1B2*, *ATP1B3* and *SLC12A2*. There were also some genes that tended to be higher expressed in the human CPE compared to the mouse CPE (*KCNA5*) or higher in mouse CPE compared to human CPE (*CLCN4*, *KCNAB2*, *KCNH1*, *KCNK3*, *SLC12A4*, *SLC4A2* and *SLC4A8*). These findings confirm previously published data on the molecular transport mechanisms in the CPE [37], [46].

**Table 4:** CPE gene expression of ion channel proteins implicated in CSF production.

Gene	H1	H2	M1	M2	M3	M4	Gene	H1	H2	M1	M2	M3	M4	Gene	H1	H2	M1	M2	M3	M4
*AQP1*	H	M	H	H	H	H	*KCNF1*	M	L	M	L	L	L	*KCNS1*	L	-	L	L	L	L
*AQP10*	L	-	-	-	-	-	*KCNG1*	L	-	-	-	M	-	*KCNS2*	L	-	L	L	L	L
*AQP11*	M	-	M	M	M	M	*KCNG2*	VL	-	-	-	M	-	*KCNS3*	M	M	L	L	M	M
*AQP12*	L	-	L	L	L	L	*KCNG3*	L	-	L	L	L	L	*KCNT1*	L	-	L	-	M	-
*AQP2*	M	-	L	M	M	M	*KCNG4*	L	-	L	M	L	L	*KCNV1*	VL	-	VL	L	L	L
*AQP3*	M	M	M	L	L	L	*KCNH1*	L	-	M	H	H	H	*KCNV2*	M	-	L	L	L	L
*AQP4*	M	L	M	M	M	M	*KCNH2*	M	L	M	M	M	H	*SCN10A*	VL	L	L	L	L	L
*AQP5*	M	-	M	L	M	M	*KCNH3*	L	-	M	L	L	M	*SCN11A*	L	L	M	L	L	L
*AQP6*	L	L	L	M	L	L	*KCNH4*	L	-	-	-	L	-	*SCN1A*	L	L	L	VL	L	L
*AQP7*	L	-	L	M	L	L	*KCNH5*	L	-	VL	-	L	L	*SCN1B*	L	L	M	L	L	-
*AQP8*	L	-	L	L	L	L	*KCNH6*	L	L	L	-	L	-	*SCN2A*	L	L	L	-	L	-
*AQP9*	L	-	L	L	L	L	*KCNH7*	M	-	L	L	L	L	*SCN2B*	L	-	M	-	L	M
*ATP1A1*	H	H	H	H	H	H	*KCNH8*	L	-	VL	VL	L	-	*SCN3A*	L	L	L	L	L	L
*ATP1A2*	H	M	H	H	H	H	*KCNJ1*	L	-	L	L	L	L	*SCN3B*	L	L	M	VL	L	M
*ATP1A3*	H	VL	M	-	M	M	*KCNJ10*	M	-	M	L	M	M	*SCN4A*	L	-	M	L	L	L
*ATP1A4*	M	-	-	-	L	-	*KCNJ11*	L	-	M	L	L	L	*SCN4B*	M	-	-	-	L	L
*ATP1B1*	H	H	H	H	H	H	*KCNJ12*	L	-	M	L	L	L	*SCN5A*	L	-	M	M	L	L
*ATP1B2*	H	M	H	H	H	H	*KCNJ13*	H	H	-	-	H	-	*SCN7A*	L	-	L	VL	L	L
*ATP1B3*	H	H	H	H	H	H	*KCNJ14*	M	L	M	M	L	L	*SCN8A*	L	-	M	L	L	L
*CLCN1*	L	-	L	L	L	L	*KCNJ15*	L	L	L	L	L	L	*SCN9A*	L	-	L	VL	L	L
*CLCN2*	M	-	M	M	M	M	*KCNJ16*	L	M	L	L	L	L	*SCNN1A*	M	M	M	M	L	M
*CLCN3*	M	M	M	M	M	H	*KCNJ2*	M	M	L	L	L	L	*SCNN1B*	L	-	M	M	L	L
*CLCN4*	L	M	H	M	H	H	*KCNJ3*	L	L	L	L	L	M	*SCNN1D*	L	-	-	-	-	-
*CLCN5*	L	L	M	L	M	M	*KCNJ4*	L	-	M	L	L	M	*SCNN1G*	VL	-	L	L	L	L
*CLCN6*	M	-	M	L	M	M	*KCNJ5*	L	VL	M	L	L	L	*SLC12A1*	L	-	M	L	L	L
*CLCN7*	M	L	M	M	M	M	*KCNJ6*	L	-	L	L	L	L	*SLC12A2*	H	M	H	H	H	H
*CLCNKA*	M	-	L	M	L	M	*KCNJ8*	H	M	M	M	M	M	*SLC12A3*	L	L	M	L	L	L
*CLCNKB*	L	-	L	M	L	L	*KCNJ9*	L	-	M	L	L	L	*SLC12A4*	M	L	H	M	H	H
*KCNA1*	VL	VL	M	L	L	M	*KCNK1*	H	M	H	H	H	H	*SLC12A5*	L	L	M	L	M	M
*KCNA10*	L	-	-	-	L	-	*KCNK10*	L	VL	-	-	L	L	*SLC12A6*	L	L	M	M	L	M
*KCNA2*	L	-	M	VL	M	M	*KCNK12*	L	-	L	L	M	M	*SLC12A7*	H	M	M	M	M	M
*KCNA3*	L	-	VL	M	M	M	*KCNK13*	M	-	L	M	L	L	*SLC12A8*	L	M	L	L	L	L
*KCNA4*	L	-	L	L	L	L	*KCNK15*	M	-	-	-	L	-	*SLC12A9*	M	L	M	M	M	M
*KCNA5*	H	M	L	VL	L	L	*KCNK16*	L	-	L	-	L	-	*SLC4A1*	L	-	M	L	L	L
*KCNA6*	L	L	M	M	L	L	*KCNK17*	L	-	-	-	-	-	*SLC4A10*	H	L	H	L	H	H
*KCNA7*	L	-	L	VL	L	L	*KCNK18*	VL	-	-	-	M	L	*SLC4A11*	-	-	L	-	M	-
*KCNAB1*	M	M	M	L	L	M	*KCNK2*	L	-	L	L	L	M	*SLC4A2*	M	L	H	H	H	H
*KCNAB2*	L	L	M	M	M	M	*KCNK3*	L	L	M	L	M	M	*SLC4A3*	M	-	M	M	M	M
*KCNAB3*	M	VL	M	M	L	L	*KCNK4*	L	-	M	L	M	M	*SLC4A4*	M	M	L	L	M	M
*KCNB1*	M	L	M	L	L	M	*KCNK5*	M	VL	M	L	L	L	*SLC4A5*	M	L	-	-	H	-
*KCNB2*	VL	VL	L	-	L	-	*KCNK6*	L	-	L	-	L	-	*SLC4A7*	M	M	M	M	M	-
*KCNC1*	L	-	M	L	L	L	*KCNK7*	M	-	M	M	L	L	*SLC4A8*	L	L	M	M	M	M
*KCNC2*	L	-	L	-	L	-	*KCNK9*	L	-	-	-	L	-	*SLC4A9*	L	-	L	L	L	L
*KCNC3*	M	-	M	M	M	M	*KCNN1*	L	VL	M	L	M	M	*SLC9A1*	L	-	M	M	M	M
*KCNC4*	L	-	L	L	M	M	*KCNN2*	M	M	M	M	L	L	*SLC9A10*	-	-	VL	-	VL	VL
*KCND1*	L	-	L	L	L	L	*KCNN3*	M	-	L	L	L	M	*SLC9A2*	L	-	L	L	L	-
*KCND2*	L	-	M	L	L	M	*KCNN4*	L	-	M	M	L	L	*SLC9A3*	VL	-	L	VL	L	-
*KCND3*	M	L	L	L	M	M	*KCNQ1*	M	-	M	M	M	L	*SLC9A5*	M	-	-	-	L	-
*KCNE1*	M	-	L	M	L	L	*KCNQ2*	L	-	VL	-	L	L	*SLC9A6*	M	M	M	L	H	H
*KCNE2*	M	-	H	M	H	H	*KCNQ3*	L	L	L	L	L	L	*SLC9A7*	L	-	L	M	M	M
*KCNE3*	M	-	L	L	L	L	*KCNQ4*	L	-	M	L	L	-	*SLC9A8*	M	-	M	M	M	M
*KCNE4*	L	L	M	M	L	L	*KCNQ5*	L	-	L	-	L	-	*SLC9A9*	M	-	L	VL	M	M

Expression levels of genes coding for ion channel proteins implicated in cerebrospinal fluid (CSF) production in healthy human and mouse CPE. We ranked the genes by expression level and assigned percentile ranks (P). Next, we formed four groups: high (H) expression (expression >90th P), moderate (M) expression (50–90th P), low (L) expression (10–50th P) and very low (VL) expression (<10th P). This means that a gene in the high expression group (>90th P) has an expression intensity that falls into the highest 10% intensity values of the microarray, whereas a gene in the very low expression group (<10th P) has an expression intensity in the lowest 10% intensity values of the microarray. For genes indicated with “-”, no data were available.

Abbreviations: H1 = our gene expression data of healthy human CPE; M1 = our gene expression data of healthy mouse CPE; H2 = healthy human CPE GSE14098; M2 = healthy mouse CPE GSE23714; M3 = healthy mouse CPE GSE37098; M4 = healthy mouse CPE GSE33009.

Functional annotation of all human and mouse CPE gene expression datasets, yielded significant functionalities implicated in *energy production and oxidative stress pathways*. Indeed, the CSF production and transport of many biomolecules over the CPE is ATP-driven and therefore the CPE requires large amounts of energy. The high energy demand of the CPE is met by the intracellular presence of a surplus of mitochondria [47], [48]. Energy producing mitochondrial pathways inevitably generate free radicals and oxidative stress.

Functional annotation of our data-driven gene expression analysis confirms that the CPEs in both men and mice are involved in *neuro-endocrine signaling*. Indeed the CPE transports many signaling molecules from the blood to the CSF, which are implicated in CSF formation, water balance, neuronal function and brain metabolism, protection, and degeneration (reviewed in [49]). Furthermore, the CPE itself also synthesizes and secretes neuro-endocrine signaling molecules (reviewed in [50]) that can regulate CSF production, for example in case of dehydration [11]. In our expression datasets of human and mouse CPE we found, for example, high expression of *APOE*, *APOD*, *BMP6*, *FGF2*, *FGF18*, *FGFR2*, *GSN*, *IGF2*, *IGFBP2*, *TGFB2*, *TTR*, *VEGFA*, and *VEGFB*. These genes were also moderately to high expressed in most of the other human and mouse CPE gene expression sub-datasets. The expression of many of these signaling molecules in both mouse and human CPE indicates that their neuro-endocrine signaling functions are also highly similar.

The analysis of our “Human and Mouse High CPE expression sub-datasets” and those of the literature, yielded several significant biological functions and canonical pathways involved in *immunological and inflammatory processes*, in viral infection, lichen planus, arthritis and auto-immune disease. Genes involved in these functionalities and highly expressed in our human and mouse CPE datasets were, for example, *ANXA5*, *−6*, *ATOX1*, *CFH*, *CD9*, *CD24*, *CD63*, *CDH11*, *HLA-B*, *HIST1H1C*, *HSPA5*, *HYAL*, *IGF2*, *IGF1R*, *IGFBP2*, *−5*, *−7*, and *ITGAV*. Indeed, according to the literature, the CPE is an important importer of T-cells and virus particles into the CNS [51]–[53] and is involved in autoimmune inflammation in multiple sclerosis [21].

In both our own and the literature's “Human and Mouse High CPE expression sub-datasets”, we annotated several significant biological functions relevant for *neurological function and disease*. Examples were AD, Parkinson's disease, and hydrocephalus. Indeed, the CPE might be involved in AD by disturbances of CSF production and composition [22]–[24], by disturbances of β-amyloid clearance [4] and breakdown of blood-CSF barrier [25]. Histological changes of the CPE in AD have been observed and included epithelial atrophy, lipofuscin vacuoles, and basement membrane thickening [19], [20] (reviewed in [54]). We also found high expression in the human and mouse CPE of the genes *CA2*, *CA12*, and *CA14*, coding for carbonic anhydrases which are involved in regulation of CSF production. Carbonic anhydrase inhibitors decrease CSF production [55] and drugs like acetazolamide, a CA inhibitor, are used as a treatment for some types of hydrocephalus [56], [57].

The *hematological function* that we found in both human and mouse CPE involved two functions: iron transport and the ability of extramedullary hematopoiesis in the CPE. Several studies suggested that the CPE is a major player in the iron homeostasis of the brain and in fact expresses all the entries known to participate in the modulation of iron homeostasis in the periphery (TFR1, DMT1, DCYTB, FPN, HJV, HFE, TFR2, FTH, FTL, TF, hephaestin, and ceruloplasmin) [8], [58]. Our gene expression data of human and mouse CPE and those of the literature corroborate this hypothesis.

Extramedullary hematopoiesis (EH) is a compensatory mechanism of hematopoiesis in other places than the bone marrow. EH is seen in patients with prolonged anemia, for example in thalassemia, sickle cell anemia, hemolytic anemia, and ineffective erythropoiesis. Some well known places for EH are the liver, spleen, and lymph nodes but also the choroid plexus was described in several human case reports [59]–[61] and in dogs [62]. Our gene expression data indicate that genes involved in hematopoiesis are indeed highly expressed in the CPE of both the human and mouse.

#### 2.2. Functions of the human CPE not annotated in mouse CPE

We did not identify human specific CPE functions that were not found in the mouse CPE.

#### 2.3. Functions of the mouse CPE not annotated in human CPE

The three major functionalities that were present in our “Mouse High CPE expression sub-dataset” and did not, or less, occur in our “Human High/Moderate CPE expression sub-datasets”, were: carbohydrate metabolism, more extensive list of (eye and neural) developmental functions and clathrin-mediated endocytosis. Analysis of comparable datasets in the literature confirmed the two first observations, but contradicted the last one. Thus, clathrin-mediated endocytosis in human and mice CPE may be less different than initially suggested by our data.

Several carbohydrate metabolism genes involved in metabolism of monosaccharide and carbohydrate and hyperglycemia (e.g. *CPT1A*, *GALK1*, *INS*, *LEPR*, *PRLR* and *SCD*) are highly expressed in mouse CPE and only lowly in human CPE. Although we can detect, due to our study design, only consistent differences between mice and men, it is not clear whether these differences are inborn or are due to a (consistent) difference in aging or diet.

The exact meaning of these findings needs further investigation.

### 3. Human and mouse specifically expressed CPE genes

#### 3.1. Human specifically expressed CPE genes

With our strict selection criteria, we did not find any human specifically expressed CPE gene compared to mouse CPE.

#### 3.2. Mouse specifically expressed CPE genes

We identified three genes specifically expressed in the mouse CPE, namely angiotensin-converting enzyme (*ACE*), serum paraoxonase/arylesterase 1 (*PON1*) and tripartite motif-containing protein 3 (*TRIM3*). These findings were partly confirmed by the other CPE gene expression datasets (Table 3).

*ACE* codes for the enzyme that converts angiotensin I to angiotensin II by release of the terminal His-Leu [63]. This leads to increased vasoconstriction and as a consequence hypertension. Inhibitors of ACE are commonly used to treat hypertension. Our findings, in combination with those of the literature, suggest that the ICP regulation via the CPE may be different in mice and humans.

PON1 is involved in the metabolism of toxic metabolites, organophosphates, lactones and aromatic carboxylic acid esters [64]. Differences in expression of *PON1* in mouse and human CPE may indicate that there are metabolic (clearance or transport) differences between the CPE of human and mouse.

Finally, TRIM3 is involved in vesicular trafficking of targeted organelles and modulation of kinesin superfamily protein (KIF) motor function [65]. This might indicate that targeted vesicle transport between the human and mouse CPE may be different.

## Conclusion

The mouse may serve well as an animal model to study diseases of the CPE. With a few single gene expression exceptions, the mouse CPE transcriptome closely resembles human CPE transcriptome. The data presented here could serve as a reference to check whether a specific gene or function of interest differs in expression between man and mouse and consequently can be studied in the mouse or not. Further protein and functional studies on single selected genes are needed to fully comprehend functional CPE differences between man and mice.

## Supporting Information

Table S1 Canonical pathways highly expressed in human and mouse CPE. This table lists all the canonical pathways assigned by Ingenuity to the “Human and Mouse High CPE expression sub-datasets”.
(DOCX)

Table S2 Functional molecular networks generated by Ingenuity of the “Human High CPE expression sub-dataset”. In this table we represent the genes involved in the 25 functional molecular networks and the top-functions assigned to these networks. We do not represent the functional relation between the genes for the sake of space reduction.
(DOCX)

Table S3 Functional molecular networks generated by Ingenuity of the “Mouse High CPE expression sub-dataset”. In this table we represent the genes involved in the 25 functional molecular networks and the top-functions assigned to these networks. We do not represent the functional relation between the genes for the sake of space reduction.
(DOCX)
