# Supplementary material for: Gene Expression and Functional Annotation of the Human and Mouse Choroid Plexus Epithelium
Source: PLoS One. 2013 Dec 31;8(12):e83345. doi: 10.1371/journal.pone.0083345 (PMC3877019; doi:10.1371/journal.pone.0083345)
Supplement: Table S1 — Canonical pathways highly expressed in human and mouse CPE. This table lists all the canonical pathways assigned by Ingenuity to the “Human and Mouse High CPE expression sub-datasets”. (DOCX) [file pone.0083345.s001.docx]

Supporting Table S1: Canonical pathways highly expressed in human and mouse CPE

| **Major functionalities** | **Components of major functionalities per species** | | |
| --- | --- | --- | --- |
|  | **Human** | **Mouse** | **Both** |
| **Actin cytoskeletal function** |  | - Actin cytoskeleton signaling  - Regulation of actin-based motility by Rho  - Actin nucleation by ARP-WASO complex | - RhoGDI signaling  - RhoA signaling  - Signaling by Rho family GTPase |
| **Epithelial junctions** |  | - Germ cell-sertoli cell junction signaling | - Remodeling of epithelial adherens junctions  - Epithelial adherens junction signaling |
| **Vesicle mediated transport** |  | - Clathrin-mediated endocytosis signaling | - Caveolar-mediated endocytosis signaling |
| **Oxidative stress** |  |  | - Mitochondrial dysfunction  - NRF2-mediated oxidative stress response  - Hypoxia signaling in the cardiovascular system |
| **Immunological function** | - Antigen presentation |  | - Protein ubiquitination |
| **Endocrine signaling/metabolism** |  | - Ephrin receptor signaling  - Aldosterone signaling in epithelial cells  - Estrogen receptor signaling  - Androgen signaling  - VEGF signaling  - Fatty acid beta-oxidation I  - TCA cycle II (Eukaryotic) | - Glucocorticoid receptor signaling  - Protein kinase A signaling |
| **Basic cellular (dys)functions** | - Aryl hydrocarbon receptor signaling  - Polyamine regulation in colon cancer | - PI3K/AKT signaling  - PTEN signaling  - ILK signaling  - Integrin signaling  - IGF-1 signaling  - 14-3-3-mediated signaling  - ERK/MAPK signaling  - Huntington’s disease signaling | - EIF2 signaling  - Regulation of eIF4 and p70S6K signaling  - mTOR signaling  - Breast cancer regulation by stathmin1 |
| **Development** |  | - Mouse embryonic stem cell pluripotency |  |
