# Supplementary material for: Gene Expression and Functional Annotation of the Human and Mouse Choroid Plexus Epithelium
Source: PLoS One. 2013 Dec 31;8(12):e83345. doi: 10.1371/journal.pone.0083345 (PMC3877019; doi:10.1371/journal.pone.0083345)
Supplement: Table S2 — Functional molecular networks generated by Ingenuity of the “Human High CPE expression sub-dataset”. In this table we represent the genes involved in the 25 functional molecular networks and the top-functions assigned to these networks. We do not represent the functional relation between the genes for the sake of space reduction. (DOCX) [file pone.0083345.s002.docx]

**Supporting Table S2: Functional molecular networks generated by Ingenuity of the “Human High CPE expression sub-dataset”**

| **Top Functions** | **Molecules in Network** |
| --- | --- |
| Hereditary Disorder, Lipid Metabolism, Neurological Disease | ARMC9, ARV1, BEX4, C10orf32, C17orf101, C18orf32, CHID1, CRLS1, CTAGE9 (includes others), CYSTM1, EFHA1, EMC10, FAM210B, FAM43B, IQCA1, JAGN1, LYRM2, MARVELD1, METTL5, MFF, MRPL21, NEMF, POLR3GL, PPDPF, RNASEH2C, RNASEK, RNF145, SLC35E4, SLC38A10, TMEM64, TMEM223, TRMT5, TSPAN10, UBC, ZNF430 |
| Gene Expression, Protein Synthesis, Molecular Transport | ALYREF, C14orf166, C22orf28, CASC3, CNOT2, CTAGE5, CUTA, DDX1, DDX3X, EBNA1BP2, EIF1AY, EIF3C/EIF3CL, EIF3D, EIF3H, EIF3K, EIF3L, EIF3M, FXR1, GRB2, HCN2, HIBCH, HIST1H2AD, HMGN3, HNRNPU, KCTD3, MYRIP, NISCH, NONO, PHACTR2, POMP, STAU1, TCEAL8, UGP2, WDR1, ZFP106 |
| Cellular Compromise, Cellular Function and Maintenance, Protein Degradation | ACTN1, ADCY9, ATXN2, BAG6, CDK11A/CDK11B, CISD2, CYB561, DERL2, EMC7, GANAB, GRM5, GUK1, HERPUD1, ITPR1, MYO1C, OS9, PJA2, PPHLN1, RAB34, RCN2, RNF103, RNF130, SEC61B, SRGN, SYVN1, TTR, UBE2Z, UBE4A, UBE4B, UBXN1, UBXN6, UFD1L, VCP, VIMP, WAC |
| Post-Translational Modification, Developmental Disorder, Hereditary Disorder | ARIH2, ATP6AP2, B3GNT9, BEX2, C4orf27, DTX3, DZIP3, FUCA1, GNL3L, IBTK, JKAMP, NFkB (complex), PNKD, RNF5, RNF13, RNF141, RNF167, SLC2A12, TMEM14C, UBE2, UBE2A, UBE2D3, UBE2E3, UBE2F, UBE2G2, UBE2L3, UBE2N, UBE2R2, UBE2V1, UBOX5, WDR34, WTAP, ZFAND5, ZFAND6, ZNF675 |
| Cellular Assembly and Organization, Cell Cycle, Cell Morphology | ABLIM1, AK2, C21orf33, CLSTN1, CTSF, CTSZ, EAPP, FAM108A1, FAM127A, FAM127B, GOLGA2, GOLGB1, KANK2, MRPS6, NAGLU, NFKBIA, OGN, PNMA1, PNMAL1, PSMA2, PSMD3, RNF181, SCFD1, SERINC1, Snare, SPG7, STX16, Ube3, UBE3A, UBQLN1, UBQLN2, USO1, VTI1B, WBP5, ZMYND10 |
| Cell Morphology, Tissue Morphology, Nervous System Development and Function | CGNL1, CSDE1, DAP3, ICT1, IFITM2, KIAA0232, LUC7L2, MALSU1, ME2, MRPL3, MRPL12, MRPL13, MRPL16, MRPL20, MRPL27, MRPL28, MRPL47, MRPL48, MRPL51, MRPL54, MRPL55, MRPS15, MRPS24, MRPS26, MRPS18A, NGRN, OXA1L, PI3K (family), PSAP, PTCD3, Shc, SLC12A2, SSFA2, STK39, WNK1 |
| Cellular Movement, Cell Morphology, Cellular Function and Maintenance | BNIP3, BNIP3L, C11orf75, COMMD1, COMMD4, COMMD6, COMMD8, Cytokeratin, DAD1, DHX9, FGF2, GLG1, GPI, GPNMB, LDHA, NCL, NDRG1, NUPR1, PA2G4, RPL3, RPL4, RPL6, RPL8, RPL18, RPL29, RPL18A, RPL7A, RPS3, secreted MMP, SLC20A1, THRAP3, TIMP2, TIMP3, TMEM11, TMX2 |
| Cancer, Hematological Disease, Metabolic Disease | B9D1, CDO1, CEBPB, CP, EEF1D, FOCAD, GARS, HTATSF1, Igh (family), P-TEFb, PDK4, PDZD2, RAD21, RPL5, RPL10, RPL13, RPL21, RPL22, RPL23, RPL28, RPL30, RPL10A, RPL13A, RPS2, RPS10, RPS11, RPS14, RPS4X, SQRDL, SSU72, STAG2, TCTN1, TMEM9, TMEM230, UBA52 |
| Developmental Disorder, Hematological Disease, Hereditary Disorder | CETN2, Collagen type XVIII, DNA-directed RNA polymerase, FAU, IGF1R, MRPL23, MRPL53, MRPS21, MYOF, PNISR, POLR2G, PRMT6, RAB18, Ribosomal 40s subunit, RPS5, RPS8, RPS12, RPS13, RPS15, RPS18, RPS20, RPS21, RPS23, RPS24, RPS26, RPS28, RPS29, RPS15A, RPS17/RPS17L, RPS27A, RPS27L, RPS4Y1, RPS4Y2, TMEM87A, ZFP36L2 |
| Cellular Development, Connective Tissue Development and Function, Cancer | BBX, C11orf58, C1orf168, CTBP1, CTBP2, Ctbp, CTNNA1, DOCK8, ENOSF1, FDFT1, GLUL, GNB1, HIPK1, HMG CoA synthase, HVCN1, KCNK1, MBTPS1, MERTK, PERP, Pias, PLA1A, PLK2, RBBP8, RGCC, RNF10, SASH1, SATB1, SIPA1L1, SLC44A1, SP100, TCF4, TMEM117, UBE2I, YPEL5, ZNF219 |
| Amino Acid Metabolism, Small Molecule Biochemistry, Cell Death and Survival | ABHD2, AIFM3, ARL1, C20orf24, CWC15, DDX5, DECR1, DPP7, DUT, EPB41L4B, Esr1-Esr1-estrogen-estrogen, EXOSC7, FLOT1, HSD17B12, LRPPRC, LRRC8A, MED4, MED13L, mediator, MPC2, MRPL41, MTHFD1, NMT1, NR3C1, OAT, PDCD7, RPLP2, RPN1, RPS25, SKIV2L2, SSR4, TM2D2, TNFAIP1, TRAP/Media, USMG5 |
| RNA Damage and Repair, Protein Synthesis, Gene Expression | Aconitase, APEX1, BZW1, CD3, CNIH4, DDT, EEF2, EEF1A1, EEF1G, FAM195A, GBAS, GLOD4, HBXIP, HMGN1, HMGN2, ILF2, ISCU, Lamin b, LONP1, NPIP (includes others), ORAI2, ORMDL3, PCYOX1, PNRC2, RBM8A, RPLP0, SDF2, SLC23A2, SMS, SRP9, SRP14, SRPRB, TARS, UPF1, ZKSCAN1 |
| RNA Post-Transcriptional Modification, Cellular Assembly and Organization, Connective Tissue Disorders | C19orf60, CCDC50, DHX15, EFTUD2, GAPDH, HNRNPA2B1, HNRNPC, IFI6, LSM3, LSM4, LSM7, LSM14A, MTORC2, NHP2L1, POP7, PRPF8, RHEB, RPS3A, SMN1/SMN2, SNRNP70, SNRNP200, snRNP, SNRPB2, SNRPC, SNRPD1, SNRPD2, SNRPD3, SNRPE, SNRPF, SNRPG, SNRPN, SNW1, STRAP, TCR, TXNL4A |
| Connective Tissue Disorders, Dental Disease, Developmental Disorder | AES, BAMBI, CNKSR3, CTNNB1, DAZAP2, DDX50, FBRS, GOLGA4, GTPBP6, HTRA1, KANK1, KIAA1109, KIF21A, LDHB, LMO2, MACF1, MAPRE1, MRPL14, MSX1, MT1L, NDN, NME3, NPHS2, PAX9, PCCA, RCN1, RNF115, SEP15, STARD7, TCF, TLE1, TMEM33, TNIK, ZFYVE9, ZIC1 |
| Cell Death and Survival, Cellular Function and Maintenance, Small Molecule Biochemistry | AGAP3, ALG8, ARL6IP4, ATP2B3, ATP2B4, ATP2C1, Ca2 ATPase, CALU, CD164, CMTM4, CRABP1, DAP, FOS, GAS5, GRB10, LETMD1, MT1A, NAE1, NBPF15 (includes others), ornithine decarboxylase, PDZD11, Pmca, POLD4, POLDIP2, Rab5, REEP5, RUFY1, SELENBP1, SLC39A13, SLC4A10, SNX3, TBCA, TRIP12, TSPYL4, ZNF467 |
| Hereditary Disorder, Metabolic Disease, Molecular Transport | ADAR, C1QBP, CCL2, COX11, COX6A1, COX6B1, COX7A1, COX7A2L, COX7B, COX7C, COX8A, CYC1, CYTB, Cytochrome bc1, cytochrome-c oxidase, ESRRA, HSPE1, lipoxygenase, MUT, NFE2L1, PAFAH1B2, PRDX3, RNASE1, RNase A, RNH1, SNUPN, UQCR10, UQCR11, UQCRB, UQCRC1, UQCRC2, UQCRFS1, UQCRH, UQCRQ, VDAC3 |
| Post-Translational Modification, Embryonic Development, Tissue Morphology | COLEC12, COPS3, COPS4, COPS5, COPS6, Cr3, CUL5, CUL4A, DARS, DCAF13, DDB1, DDIT4, EMG1, EPRS, FOXP1, HOPX, ITGAM, KLHL9, KLHL21, MAP7D1, MARS, MYEOV2, NEDD8, PDPN, PTRF, PWP1, Rag, RARS, RNA polymerase I, RNF7, SCAVENGER receptor CLASS A, SHISA5, WSB1, WSB2, ZNF148 |
| RNA Post-Transcriptional Modification, Gene Expression, Protein Synthesis | 60S ribosomal subunit, CNBP, ERH, G protein, Gap, Gi-coupled receptor, HINT1, HNRNPR, PABPC3, RPL9, RPL14, RPL15, RPL17, RPL19, RPL24, RPL26, RPL27, RPL32, RPL34, RPL36, RPL37, RPL38, RPL39, RPL41, RPL26L1, RPL27A, RPL35A, RPL36A, RPL36AL, RPL37A, RPS16, RPS19, SARNP, SRRT, ZC3H11A |
| Post-Translational Modification, Cell Morphology, Cell-To-Cell Signaling and Interaction | AHCYL1, Ap1, BECN1, BMI1, C19orf70, CSNK1E, DLC1, DUB, FUNDC2, GLRX3, I kappa b kinase, ITFG1, MT2A, NACA, PHC2, PIK3C3, PTOV1, Smad2/3, SNTA1, SNTB2, TAGLN2, TCEAL2, TCEAL4, TMEM70, TNFRSF14, USP1, USP4, USP11, USP16, USP39, USP48, USP9X, UTRN, ZNF24, ZNHIT3 |
| RNA Post-Transcriptional Modification, Cellular Movement, Connective Tissue Development and Function | ACO2, AMD1, BLVRA, DDX17, DGCR6, G-Actin, HNRNPA1, HNRNPA1L2, HNRNPF, HNRNPH1, HNRNPH2, HNRNPH3, HNRNPL, HNRNPM, HNRNPUL2, HNRPDL, JUN, MBNL1, PABPN1, PXDN, RGPD5 (includes others), SERP1, SLC30A1, SRP19, SRRM2, TARDBP, Tcf 1/3/4, TMC8, TMSB4, TMSB10/TMSB4X, TNPO1, TPM2, Transportin, UBIQUITIN LIGASE, YTHDF2 |
| Hereditary Disorder, Metabolic Disease, Cardiovascular Disease | AIP, amylase, ANXA11, ECHDC1, MAGED1, Mapk, NADH2 dehydrogenase, NADH2 dehydrogenase (ubiquinone), ND2, ND3, ND5, NDUFA1, NDUFA2, NDUFA4, NDUFA5, NDUFA8, NDUFA9, NDUFA10, NDUFA11, NDUFB2, NDUFB4, NDUFB6, NDUFB7, NDUFS4, NDUFS5, NDUFS6, NDUFS8, NDUFV1, NDUFV2, PEG3, SDF4, Tenascin, TPD52, TPD52L1, WIPI2 |
| Amino Acid Metabolism, Nucleic Acid Metabolism, Small Molecule Biochemistry | ACTL6A, ACTR6, C17orf79, CDR2, GUSB, H2AF, H2AFJ, H2AFV, H2AFY, H2AFZ, HIST2H2AC, INO80D, MAN1C1, MAN2B1, Mannosidase Alpha, MAT2A, MAT2B, MEAF6, MLF2, MORF4L1, MORF4L2, MPDU1, MRFAP1, MRFAP1L1, PARP10, PITPNA, PMVK, RAB3IP, Ras, SMAD1/5, TIP60, TRAPPC4, TRAPPC5, VEZF1, ZNHIT1 |
| RNA Post-Transcriptional Modification, Developmental Disorder, Hereditary Disorder | ARGLU1, C19orf43, Cebp, CIRBP, CTSA, FUS, HDGF, HIST2H2AA3/HIST2H2AA4, HNRNPA3, LBX1, MALAT1, NEU1, P38 MAPK, PKC alpha/beta, PNN, PUF60, RBM39, RBMX, SF1, SF3A2, SF3B1, SF3B5, SF3B14, Srp30, SRRM1, SRSF1, SRSF2, SRSF3, SRSF4, SRSF5, SRSF9, TRA2B, U2AF1, U2af, ZRANB2 |
| Cell Death and Survival, Cancer, Endocrine System Disorders | Alpha tubulin, BBS5, Beta Tubulin, CCT2, CCT3, CCT4, CCT5, CCT7, CCT8, CCT6A, CLTA, DSP, FAM82A2, GCN1L1, Integrin alpha 2 beta 1, Integrin alpha 3 beta 1, KIFAP3, PFDN2, PFDN5, PFDN6, PPP2CB, Rac, SLC3A2, SLC7A8, STK24, SYNE1, TCP1, TMEM109, TUBA1A, TUBA1C, TUBA3C/TUBA3D, TUBB4B, VAPA, WDR82, ZFYVE27 |
| Cellular Assembly and Organization, DNA Replication, Recombination, and Repair, Nucleic Acid Metabolism | BRD2, C16orf53, CBX1, CBX3, CBX5, CBX7, CTR9, DPY30, E2F6, EID1, FBL, G-protein beta, G3BP2, GRSF1, H3F3A/H3F3B, HAT1, Hat, HIST1H3A (includes others), HIST1H4A (includes others), HIST3H3, HIST3H2A, ITGB1BP1, MATR3, Mi2, MLL3, NDPK, NME1, NME2, NOP58, NuRD, PGAM1, PROS1, RBBP7, STOM, WWC1 |
