# Supplementary material for: Gene Expression and Functional Annotation of the Human and Mouse Choroid Plexus Epithelium
Source: PLoS One. 2013 Dec 31;8(12):e83345. doi: 10.1371/journal.pone.0083345 (PMC3877019; doi:10.1371/journal.pone.0083345)
Supplement: Table S3 — Functional molecular networks generated by Ingenuity of the “Mouse High CPE expression sub-dataset”. In this table we represent the genes involved in the 25 functional molecular networks and the top-functions assigned to these networks. We do not represent the functional relation between the genes for the sake of space reduction. (DOCX) [file pone.0083345.s003.docx]

**Supporting Table S3: Functional molecular networks generated by Ingenuity of the “Mouse High CPE expression sub-dataset”**

| **Top Functions** | **Molecules in Network** |
| --- | --- |
| Cellular Assembly and Organization, Cellular Function and Maintenance, Hereditary Disorder | ABHD14B, ALG14, C16orf13, C17orf101, C18orf32, C8orf59, CLIP3, CNPPD1, EFHA1, EMC10, FAM127C, FAM168B, FAM195B, IFT46, JAGN1, KIAA0100, KLHDC8B, MFF, MORN1, PPDPF, RBM27, RNASEH2C, RNASEK, RPP25L, SLC38A10, SNX25, SYS1, TBC1D13, TMEM64, TMEM223, TMEM167A, UBC, UPK3BL, VPS53, ZNF28 |
| Developmental Disorder, Hereditary Disorder, Metabolic Disease | ATG12, Cbr2, CNBP, CRIP2, CTSZ, ERBB2IP, FAM213A, FLRT2, GOLGA7, HNRNPA3, HNRNPAB, HNRNPUL1, HRAS, Ifi27l1, IFITM2, MAGOH, MFGE8, MSMO1, MSRB1, NUCKS1, OXCT1, PCYT2, PDZD2, PIGS, PIGT, PUM2, Rps17, SESN3, SPIN1, SQLE, TINAGL1, TKT, TRAPPC12, UPF3A, ZDHHC9 |
| Hematological Disease, Hereditary Disorder, Cell Morphology | AKIRIN1, ALKBH5, AMMECR1L, ANO6, C19orf42, C22orf25, C5orf15, C6orf62, C6orf106, CASC4, CASD1, CCDC19, CDR2L, DRAM2, ELAVL1, FAM114A2, FAM73A, HERPUD2, KIAA0494, KIAA1737, KIAA2013, MEX3C, MPV17L, PCMTD1, SLC18B1, SLC48A1, SPPL3, TM7SF3, TM9SF3, TMCO3, TMEM127, TMEM134, TMEM63B, TSPAN31, WDR45L |
| Developmental Disorder, Hereditary Disorder, Neurological Disease | ATP6AP2, BEX2, CCT2, CCT5, CCT7, CCT8, FUCA1, JKAMP, LUC7L3, MYO1E, NFkB (complex), PAK1IP1, PNKD, PPP4C, PPP4R2, PPP6C, RAB3IP, RIOK3, RNF5, SLC2A12, SMEK1, SSR4, TMEM14C, TRAPPC3, TRAPPC4, TRAPPC5, TRAPPC9, TRAPPC2L, TRAPPC6A, TRAPPC6B, TRPC4AP, VMP1, WDR34, WTAP, ZNF385A |
| Developmental Disorder, Hereditary Disorder, Metabolic Disease | AP4S1, ARSG, C14orf166, C22orf28, CD164, CLYBL, CTAGE5, CUTA, DLGAP4, EBNA1BP2, GNS, GRB2, HELZ, IDS, MSI2, MYRIP, NADK, PEX13, PHACTR4, POMP, PURA, PURB, SLC25A1, SLC4A2, SNX3, SNX17, SPRY, SS18, SULF1, SUMF1, TCEAL8, UGP2, WDR1, XRN2, ZFP106 |
| Cell Morphology, Cellular Function and Maintenance, Developmental Disorder | ADSL, AKIP1, ASCC3, ATG4A, CLNS1A, EIF5B, FARS2, FNIP1, FUNDC1, FYCO1, G3BP1, G3BP2, GABARAP, GABARAPL1, GABARAPL2, GFPT1, MRPL45, MTDH, NMT1, OTUD6B, PAICS, Pki, PKIG, PRKACA, PRKAG1, SIK3, SND1, TBC1D9, TBC1D14, TMEM160, TP53INP2, UBAP2, ULK1, USP10, ZNF706 |
| RNA Post-Transcriptional Modification, Protein Synthesis, Cell-mediated Immune Response | ACO2, AHCYL1, AHCYL2, BCAS2, DPP3, GNB2L1, GTF3C6, HNRNPL, ICT1, ISCA1, JOSD2, KHDRBS3, LARP4B, MRPL12, MRPL13, MRPL18, MRPL24, MRPL43, MRPS15, MRPS26, MRPS34, PGRMC1, PNKP, PPM1G, PTCD3, RALY, Rasgrf, RBM7, RPS7, SF3B2, SRRM2, TRUB2, UBE2O, YBX1, ZNF579 |
| Hereditary Disorder, Metabolic Disease, Cell Morphology | APRT, C1QBP, CYC1, Cytochrome bc1, cytochrome-c oxidase, DHRS7B, ECH1, ESRRA, HSPE1, LMAN1, MAPK3, MCFD2, NFE2L1, OPA1, PAM16, PCBP3, SF3A3, TIMM10, TIMM22, TIMM44, TIMM17A, TIMM17B, TIMM8B, TOMM6, TOMM7, UQCR10, UQCR11, UQCRB, UQCRC1, UQCRC2, UQCRFS1, UQCRH, UQCRQ, VDAC3, YME1L1 |
| DNA Replication, Recombination, and Repair, Energy Production, Nucleic Acid Metabolism | ABCA2, ABCD3, ATAD1, ATP1B1, ATPase, CD3, COX7A1, FYTTD1, GTF2IRD1, HNRNPR, HSP90AA1, IDE, KATNA1, KIF1B, LONP1, MACF1, MRPL11, MYH7, MYH9, NAGA, NIT1, OGN, ORAI3, PEX6, S100A11, SLC23A2, SLC5A3, STEAP2, SYAP1, TMX3, TPST1, TSPAN7, TSPAN9, UBE4A, ZFP36L1 |
| Cell Morphology, Cell Cycle, Cellular Assembly and Organization | AKT1, AKTIP, B3GAT3, BET1L, C1QC, CLSTN1, COG7, DENR, DNM1L, FAM108A1, FIS1, GOLGA2, GOSR1, GOSR2, GPC4, MARCH5, MCTS1, ME1, METAP2, MFN1, MFN2, PEX11B, RNF123, SEC22B, SLC35B2, Snare, STX7, STX18, THYN1, TRIM41, UBAC1, USE1, VAMP7, VTI1B, YKT6 |
| Cellular Assembly and Organization, Cellular Function and Maintenance, Post-Translational Modification | AAR2, ARF3, ARF6, ARFGEF2, BFAR, DAD1, GSPT1, HDLBP, HYOU1, IQSEC1, LMAN2, MGAT2, MTHFD1, OAT, PCBD2, PDIA4, Pik3r, RCC2, RCN3, Rrbp1, SDF2L1, SEC13, SEC11A, SEC11C, SELM, signal peptidase, SPCS1, SPCS2, SPCS3, SRP9, SRP14, SSR1, TALDO1, XBP1, YIPF5 |
| Gene Expression, Protein Synthesis, Developmental Disorder | ANXA3, CETN2, CETN3, DNA-directed RNA polymerase, EMG1, FAU, IGF1R, MRPL23, MRPL40, MRPL53, MRPS21, MYOF, NAT6, PDE6 (rod), PDE6D, POLR2G, RAB18, Ribosomal 40s subunit, RPS5, RPS8, RPS11, RPS12, RPS13, RPS15, RPS18, RPS21, RPS23, RPS24, RPS26, RPS27, RPS29, RPS15A, RPS27L, RPS4Y1, SF3B5 |
| Protein Degradation, Protein Synthesis, Cellular Compromise | AMFR, AUP1, CLDN1, CLDN2, CLDN3, cldn, DERL1, DERL2, DFNB31, ERLEC1, ERLIN2, FAF2, Gsk3, GUK1, Guk, HERPUD1, MPP1, NSFL1C, OS9, PJA2, RNF103, RNF130, RNF166, RPN1, SEL1L, STT3B, SYVN1, TJP2, UBE2Z, UBR2, UBR3, UBXN1, UBXN6, VCP, VIMP |
| Cellular Assembly and Organization, Cell Death and Survival, Cell Morphology | 2210013O21Rik, AAMP, ARMCX3, BAG6, C8orf33, CCDC113, CHCHD3, CHCHD6, CRELD2, CRIP1, DEPTOR, EPHX1, epoxide hydrolase, FAM173A, GPRASP1, Granzyme, HBXIP, HMOX2, IL12 (family), IMMT, IQCG, ITFG1, MAF1, MEST, MIA3, MINOS1, MRFAP1, MTX1, MTX2, NOL7, RER1, SAMM50, SRGN, TXNDC9, ZC3H15 |
| Post-Translational Modification, Amino Acid Metabolism, Cell Death and Survival | AHSA1, ARL1, ATXN10, BAIAP2L1, C11orf83, C20orf24, CCDC47, CLPP, DDOST, FAM96B, GSTK1, HEATR2, IPO11, KRTCAP2, Ly6a (includes others), MPC2, MRPL41, MRPS10, MRPS16, NDFIP2, PHLDA3, RFC2, Rfc, RPA, RPN2, SCOC, SEC62, SEC63, SLC19A1, SRC (family), TMBIM4, TMCO1, TMEM11, UNC45A, USMG5 |
| Connective Tissue Disorders, Developmental Disorder, Hereditary Disorder | 14-3-3(β, γ, θ, η, ζ), 14-3-3(η, θ, ζ), C16orf80, C1orf63, CDK2AP2, CGNL1, CHP1, CLK3, CYFIP1, DCAF7, DOCK7, FAM13B, FRYL, FXR1, GNB1, HMG CoA synthase, HMGCS1, KIAA1598, KIF1C, LARP1, LSR, MBTPS1, N4BP3, PPFIBP1, PRSS23, RASSF8, SERBP1, SON, TDRD3, TMEM222, WNK1, YIF1A, YWHAB, YWHAG, YWHAH |
| Cancer, Hematological Disease, Dermatological Diseases and Conditions | 60S ribosomal subunit, CAPRIN1, CIC, endothelin receptor, G protein alpha, GLOD4, GNA13, GNAI3, HNRNPU, IVNS1ABP, NUCB1, NUCB2, OLA1, PIN4, PNRC2, PRMT1, RBM17, RBMXL1, RGS12, RPL3, RPL4, RPL6, RPL8, RPL37, RPL39, RPL22L1, RPL36A, RPL37A, RPL7A, RPL7L1, SF1, SNURF, SPAG9, THRAP3, ZNRD1 |
| Cellular Function and Maintenance, Small Molecule Biochemistry, Molecular Transport | ADSSL1, AIFM1, ATOX1, ATP7A, AZIN1, BNIP3, C16orf58, CAPN7, CAPNS1, CNPY3, COMMD1, COMMD3, COMMD6, COMMD7, COMMD10, CREB3, DDIT4, DOLK, DPP8, GNB2, GPX4, GRHPR, LPCAT2, MTORC2, OAZ1, OAZ2, OAZ, RHEB, SYNGR1, TECR, TMEM9, TMEM230, TMEM234, Tsc1-Tsc2, UNC50 |
| Post-Translational Modification, Protein Degradation, Protein Synthesis | CBX6, Clathrin, DUB, ERP44, H3f3a/H3f3b, HAT1, Hat, Hist2h4 (includes others), HIST3H2BB, HUWE1, IARS, KLHDC10, LARS, MYO6, NEDD8, PDLIM2, QARS, RNF11, RPL12, RPL10A, TAX1BP1, TBCB, TP53BP2, UBA52, USP2, USP4, USP5, USP7, USP16, USP25, USP32, USP33, USP34, WRNIP1, ZNF207 |
| Carbohydrate Metabolism, Ophthalmic Disease, Metabolic Disease | 6-phosphofructokinase, CA2, CA12, CA14, CACYBP, Carbonic anhydrase, CCNDBP1, CUX1, DNAL4, DYNLL1, DYNLL2, ELOVL7, FAM107B, GPBP1, GPRC5B, HNRNPA2B1, IMP3, LSM4, LSM6, LSM7, MLF1, NGFRAP1, NRBP1, PFKL, PFKM, PFKP, phosphofructokinase, RPL13, RPL28, RPL30, RPL13A, RPLP0, SRI, TXNDC17, UHRF1BP1L |
| Connective Tissue Disorders, Dermatological Diseases and Conditions, Developmental Disorder | ACAT1, ADCK4, BBX, BRD2, CCAR1, COMT, DAP3, DEGS1, DPF2, DPP7, DUT, Eotaxin, GADD45, HPRT1, HSD17B12, HSD17B, IFT122, LMO4, LRRC8A, NR3C1, PDCL3, PTMS, PTP4A1, PTP4A2, SASH1, SLC16A1, SLC38A1, Tgtp1/Tgtp2, TM2D2, TM2D3, TNFAIP8, TPST2, WDR6, WDR19, ZHX1 |
| Cellular Movement, Connective Tissue Development and Function, Developmental Disorder | AMD1, API5, CRELD1, DHRS3, EEF1B2, EEF1D, EEF1G, FAM195A, FBXW11, HDC, LPGAT1, MAOB, Mre11, MSRB2, MT3, PDGF (family), PHLDA1, RAD21, RAF1, RPS2, RPS3, RPS10, RPS14, RPS16, RPS19, RPS3A, SETD3, SSU72, ST6GALNAC2, TCF25, TFG, TMSB4, TMSB10/TMSB4X, XRCC5, YPEL5 |
| Gene Expression, Embryonic Development, Organ Development | AES, ARL6IP5, BCL2L1, BTF3, Cbp/p300, CDK9, CISD2, COBRA1, DAZAP2, DHX9, EDF1, ENO1, GSR, GTF2A2, GTF2F1, GTF2H5, Holo RNA polymerase II, IRF, KIAA1967, MAGED1, MSX1, PEF1, PJA1, POLR2C, POLR2E, POLR2F, POLR2I, POLR2K, ROMO1, RPL18A, RTN1, SETD1B, TAF10, TLE1, WDR82 |
| Free Radical Scavenging, Energy Production, Nucleic Acid Metabolism | ATP9A, CLIC4, COX4I1, COX5A, COX5B, COX6B1, COX6C, COX7A2, CYB5B, CYCS, cytochrome C, dolichyl-diphosphooligosaccharide-protein glycotransferase, FAM162A, FBXO33, FDX1, FTSJD2, HK1, HSP90AB1, HSPD1, IARS2, IRF2BP2, Lamin, MCL1, MPST, MRPS12, MTCH1, PHB, PHF3, PRKAR1A, SLC25A17, SLCO1C1, SOD1, TUSC3, TXN2, VEGFA |
| Developmental Disorder, Hereditary Disorder, Drug Metabolism | ARPP19, ATF5, B9D1, C11orf54, CEBPB, CIRBP, CYP19, Cytokeratin, ECI1, ELOVL1, EXOSC5, GPI, HRSP12, HSD17B11, HSDL2, Ifitm3, INMT, KIAA1217, KRT15, LGALSL, NDRG4, PDK4, POR, RETSAT, SQRDL, TCTN2, TMEM17, TMEM176A, TMEM184C, TMEM229B, TMEM50B, TNFRSF19, UGT, UGT1A6, WAC |
